# Metabolic syndrome and risk of advanced colorectal adenomas in a screening population: Frequentist and Bayesian analyses

**DOI:** 10.1111/codi.70424

**Published:** 2026-03-19

**Authors:** Franz Singhartinger, Georg Semmler, Vera Paar, Michael Lichtenauer, Andreas Völkerer, Josef Holzinger, Mathias Ausserwinkler, Maria Flamm, Elmar Aigner, Christian Datz, Bernhard Wernly

**Affiliations:** ^1^ Department of Surgery Paracelsus Medical University Salzburg Austria; ^2^ Division of Gastroenterology and Hepatology, Department of Medicine III Medical University of Vienna Vienna Austria; ^3^ Department of Internal Medicine II Paracelsus Medical University Salzburg Austria; ^4^ Hospital Oberndorf Oberndorf Austria; ^5^ Department of Internal Medicine 1 Paracelsus Medical University Salzburg Austria; ^6^ Department of Internal Medicine Elisabethinen Hospital Klagenfurt Klagenfurt Austria; ^7^ Institute of General Practice Family Medicine and Preventive Medicine, Paracelsus Medical University Salzburg Austria

**Keywords:** adenoma, colon cancer, leptin, metabolic syndrome, screening colonoscopy

## Abstract

**Background:**

Screening colonoscopy prevents colorectal cancer (CRC) by detecting and removing premalignant lesions. Metabolic syndrome (MetS) has been proposed as an additional risk marker to refine risk stratification beyond age‐based screening, but its independent association with advanced colorectal neoplasia remains uncertain.

**Methods:**

We conducted a cross‐sectional analysis of asymptomatic adults undergoing screening colonoscopy in the Salzburg Colon Cancer Prevention Initiative. Analyses were limited to participants with complete‐case data for the Adult Treatment Panel III (ATP III) MetS definition (*N* = 4891). The primary endpoint was advanced colorectal lesions, defined as advanced adenoma and/or CRC. We estimated incidence rate ratios (IRR) using Poisson regression with robust variance in unadjusted, age/sex‐adjusted and fully adjusted models (demographic and lifestyle covariates). Sensitivity analyses applied the International Diabetes Federation (IDF) MetS definition and insulin resistance (HOMA‐IR; binary and per doubling). In an exploratory subsample, leptin (per doubling) was evaluated, including joint models with MetS. Bayesian models (non‐informative, pessimistic, sceptical priors) quantified posterior effect distributions and equivalence probabilities.

**Results:**

Advanced lesions occurred in 389/4891 participants (7.95%) and were more frequent in ATP III MetS (9.66% vs. 6.97%; *p* = 0.001). In frequentist analyses, ATP III MetS was associated with advanced lesions in unadjusted models (IRR: 1.39; 95% CI, 1.15–1.68) but not after age/sex adjustment (IRR: 1.09; 95% CI, 0.90–1.32) or full adjustment (IRR: 1.06; 95% CI, 0.83–1.36). Age was the dominant predictor (fully adjusted IRR: 1.73 per decade; 95% CI, 1.53–1.95), while female sex was protective (IRR: 0.57; 95% CI, 0.44–0.73). Findings were concordant using the IDF definition (unadjusted IRR: 1.39; age/sex‐adjusted 1.09; fully adjusted 1.07) and for HOMA‐IR (binary: no association; per doubling: unadjusted IRR: 1.10, *p* = 0.052; adjusted null). In the leptin subsample (*N* = 602), leptin was not independently associated with advanced lesions (per doubling IRR: 0.85 unadjusted; 0.96 age/sex‐adjusted; 0.95 fully adjusted) and did not materially alter MetS estimates. Bayesian analyses mirrored attenuation with adjustment; in fully adjusted models, sceptical priors yielded a posterior median IRR of 1.01 (95% CrI: 0.91–1.12) with a 92.9% probability that the MetS effect lay within ±10% of no effect.

**Conclusions:**

The apparent excess risk of advanced colorectal lesions in MetS is explained by age and sex. Across MetS definitions, HOMA‐IR, leptin and Bayesian sensitivity analyses, MetS does not provide independent incremental information for CRC screening risk stratification beyond established demographic factors.

**What does this paper add to the literature?**

This research presents the first Bayesian analysis examining the relationship between metabolic syndrome and the risk of advanced adenomas within a Caucasian screening cohort. Additionally, it is the first to assess the association between leptin levels and the likelihood of developing advanced adenomas.

## INTRODUCTION

Colorectal cancer (CRC) is the third most commonly diagnosed malignancy worldwide and the second leading cause of cancer‐related death, with nearly two million new cases and close to one million deaths annually [1]. Screening colonoscopy effectively prevents CRC by detecting and removing adenomatous polyps, yet current recommendations rely almost exclusively on age‐based thresholds, adjusted for gender, typically beginning between ages 45 and 50 years [2, 3].

This age‐based strategy does not account for the substantial heterogeneity in adenoma risk observed within age groups depending on lifestyle and probably other unknown factors [4, 5]. Identifying additional risk factors could refine screening approaches and improve the balance of benefits and harms.

Metabolic Syndrome (MetS), a constellation of abdominal obesity, dyslipidaemia, hypertension and impaired glucose metabolism, is common in Western populations. It has been implicated in carcinogenesis through pathways involving insulin resistance, chronic inflammation and altered adipokine signalling [6].

Epidemiological studies have reported associations between individual MetS components and colorectal neoplasia, but evidence for MetS as a composite predictor remains inconsistent [7, 8, 9, 10]. Prior studies have generally adjusted for age and sex, yet the extent to which MetS provides risk information beyond these dominant demographic factors remains uncertain. Given that both MetS prevalence and adenoma risk increase steeply with age and differ substantially between men and women, disentangling shared demographic effects from potential metabolic mechanisms is crucial for clarifying the clinical relevance of MetS in colorectal carcinogenesis.

Leptin, a key adipokine reflecting adiposity and insulin resistance, has been implicated in colorectal carcinogenesis through pro‐inflammatory, pro‐proliferative and anti‐apoptotic pathways in experimental models. However, epidemiological evidence linking circulating leptin to colorectal neoplasia remains limited and inconsistent [11, 12, 13]. We therefore examined the association between MetS and colorectal adenoma risk in a large screening cohort, with a particular focus on advanced adenomas, the lesions most relevant for cancer prevention [14, 15, 16]. In addition, we explored whether circulating leptin provides independent or complementary risk information. This might be due to the promotion of chronic low‐grade inflammation and oxidative stress exacerbating MetS pathophysiology [17]. To address uncertainty and assess robustness, we applied both frequentist and Bayesian statistical approaches.

## METHODS

### Study population and design

We conducted a cross‐sectional analysis within the Salzburg Colon Cancer Prevention Initiative (SAKKOPI), a prospective registry of asymptomatic individuals undergoing screening colonoscopy at the General Hospital Oberndorf, Austria, between January 2007 and March 2020. Participants were recruited via referral by primary care physicians or by self‐enrolment in an opportunistic, insurance‐funded screening program providing colonoscopy at no cost. SAKKOPI reflects an opportunistic screening setting and was not based on an organized invitation strategy. Although faecal tests (e.g. FIT) may be used in routine primary care, FIT results were not systematically recorded in the registry and were not used to determine eligibility for screening colonoscopy within SAKKOPI. Colonoscopies were performed under a screening indication; gastrointestinal symptoms were not recorded in a standardized manner, and some participants may have had non‐specific complaints at the time of referral or self‐enrolment. Austrian guidelines recommend screening colonoscopy from the age of 50 years for asymptomatic adults. All participants provided written informed consent for the scientific use of their data. Clinical and laboratory data were collected using standardized procedures, including structured questionnaires (medical history and lifestyle) and anthropometric measurements performed by trained personnel. The local Ethics Committee for the province Salzburg approved the study protocol (approval no. 415‐E/1262). Written informed consent was obtained from every participant.

### Eligibility and analytical samples

The source population comprised all individuals who underwent screening colonoscopy within SAKKOPI during the study period (*N* = 6368). The main analytical sample for the primary analyses included participants with complete data required to define MetS according to Adult Treatment Panel III (ATP III) criteria and to ascertain the colonoscopy outcomes (complete‐case cohort; *N* = 4891) [18]. Participants were excluded from the main analytical sample if any required variable was missing (*N* = 1477). A comparison of included and excluded participants is provided in Table S1. Because our primary exposure (ATP III MetS) and fully adjusted models require complete information across multiple clinical and questionnaire‐derived variables, we used a complete‐case approach and did not impute missing values.

### Definition of metabolic syndrome

MetS was defined according to ATP III criteria as the presence of ≥3 of the following components: (1) abdominal obesity (waist circumference >102 cm in men or >88 cm in women); (2) elevated triglycerides (≥150 mg/dL or lipid‐lowering therapy); (3) low HDL cholesterol (<40 mg/dL in men or <50 mg/dL in women or lipid‐lowering therapy); (4) elevated blood pressure (≥130/85 mmHg or antihypertensive therapy); and (5) elevated fasting glucose (≥100 mg/dL or glucose‐lowering therapy). In sensitivity analyses, MetS was additionally defined using the International Diabetes Federation (IDF) criteria. The IDF definition requires central obesity (waist circumference ≥94 cm in men or ≥80 cm in women for Europeans) plus ≥2 of the following: (1) elevated triglycerides (≥150 mg/dL or treatment); (2) low HDL cholesterol (<40 mg/dL in men or < 50 mg/dL in women or treatment); (3) elevated blood pressure (≥130/85 mmHg or treatment); (4) elevated fasting glucose (≥100 mg/dL or treatment). Insulin resistance was assessed using HOMA‐IR and evaluated both as a binary indicator (HOMA‐IR >2.5) and as a continuous measure scaled per doubling (ln[HOMA‐IR]/ln [2]).

### Outcomes

The primary outcome was advanced colorectal lesions, defined as the presence of advanced adenoma and/or CRC. Advanced adenoma was defined as ≥1 adenomatous polyp with any high‐risk feature: diameter ≥10 mm, villous/tubulovillous histology or high‐grade dysplasia. Secondary outcomes included any adenoma and adenoma multiplicity, based on colonoscopy findings and histopathology reports.

### Leptin measurement and handling

In an exploratory subsample (*n* = 602) with available biospecimens, serum leptin was measured using a standard enzyme‐linked immunosorbent assay (ELISA; R&D Systems, Minneapolis, MN, USA). Leptin measurements were available only for participants enrolled in the most recent phase of the registry (up to 2020), reflecting an extension of the biobanking protocol that was intended for ongoing future sampling; this component was discontinued when study operations were curtailed in the context of the COVID‐19 pandemic. Leptin was analysed as a continuous exposure after natural log‐transformation and scaling by ln(2), enabling interpretation per two‐fold increase in leptin concentration. In secondary exploratory analyses, leptin was additionally categorized by the cohort‐specific median. Leptin analyses were conducted within the subset of participants with available leptin measurements and required covariate data for the respective models. The leptin subsample consisted of participants with available biospecimens from the registry's most recent phase and therefore represents a non‐random subset of the overall screening cohort. Baseline characteristics and colonoscopy findings of participants with available leptin measurements, stratified by ATP III MetS status, are provided in Table S2. Regression analyses in the leptin subsample were conducted as complete‐case per model.

### Statistical analysis

Baseline characteristics were summarized as medians (interquartile ranges) for continuous variables and counts (percentages) for categorical variables. Group comparisons used the Wilcoxon rank‐sum test or *χ* ^2^ test, as appropriate. Associations between exposures and binary outcomes were estimated using Poisson regression with a log link and robust (sandwich) standard errors, reporting incidence rate ratios (IRR) with 95% confidence intervals. Three models were prespecified: (1) unadjusted; (2) adjusted for age (per decade) and sex; and (3) fully adjusted for smoking status, alcohol consumption, family history of CRC, educational level, diet quality and physical activity. Regression analyses were conducted as complete cases for the respective model. We prespecified an unadjusted model and an age/sex‐adjusted model as the primary confounding assessment, followed by an extended multivariable model including lifestyle and sociodemographic covariates to evaluate robustness beyond the dominant demographic determinants. Model‐specific analytic sample sizes are reported in Table 3. Multiplicative interaction terms were used to assess effect modification. Accordingly, the analytical sample size may differ across models depending on covariate availability; denominators are reported with the respective model outputs. As exploratory analyses, we evaluated metabolic burden beyond a binary MetS definition by modelling the number of ATP III components (0–5) as a continuous/ordinal exposure (per additional component) in Poisson regression with robust standard errors, using the same adjustment strategy as in the primary models. Missing data were handled using complete‐case analysis. The primary analytic cohort (*N* = 4891) included all participants with complete information to define ATP III MetS and to ascertain colonoscopy outcomes. Multivariable models requiring additional lifestyle and sociodemographic covariates were estimated in correspondingly reduced samples with complete covariate data (see Table 3 for model‐specific denominators).

### Bayesian analysis

Bayesian models complemented the frequentist analyses for the MetS effect. Three prior strategies were specified for the MetS coefficient: (1) non‐informative Normal (0, 100^2^); (2) pessimistic Normal (ln[1.10], 0.058^2^), anticipating a 10% increased risk; and (3) sceptical Normal (0, 0.058^2^), centred on the null. Posterior probabilities were derived for clinically relevant thresholds (IRR >1.0, >1.05, >1.10) and for regions of practical equivalence (ROPE) defined a priori as ±5% and 10% around IRR = 1.0. Convergence was assessed using R‐hat statistics (<1.01) and visual inspection of trace plots. Posterior medians and 95% highest posterior density credible intervals (CrI) are reported.

## RESULTS

Among the 4891 participants in the primary analytic cohort, 1790 (36.6%) met ATP III criteria for MetS, while 3101 did not. Participants with MetS were older (median age 61 vs. 55 years), more frequently male (59% vs. 47%), and had lower educational attainment (lower education 38% vs. 31%; high education 7% vs. 11%) (Table 1). As expected, adiposity and cardiometabolic parameters differed substantially between groups, including higher body mass index (29 vs. 25 kg/m^2^) and waist circumference (104 vs. 91 cm), higher blood pressure, higher fasting glucose and HbA1c and lower HDL cholesterol in the MetS group (Table 1). Lifestyle profiles also differed, with more frequent ever smoking (52% vs. 46%), higher alcohol consumption ≥2 drinks/day (14% vs. 10%) and less favourable distributions of physical activity and diet quality among participants with MetS (Table 1). Compared with included participants, excluded individuals (*n* = 1477) were older and exhibited a markedly less favourable metabolic and lifestyle profile, including higher adiposity, higher blood pressure and glycaemic measures and a substantially higher prevalence of MetS and its individual components (Table S1). Notably, colonoscopy outcomes were similar between excluded and included participants, including advanced lesions (8% vs. 8%, *p* = 0.68) and any adenoma (31% vs. 33%, *p* = 0.38), suggesting limited selection‐related distortion of endpoint distributions (Table S1).

**TABLE 1:** Baseline characteristics of participants by metabolic syndrome status.

Characteristic	No MetS (<3 criteria)	MetS (≥3 criteria)
	*N* = 3101	*N* = 1790
Demographics		
Age, years	55 (51–63)	61 (54–69)
Male sex	1472 (47%)	1048 (59%)
Educational level		
Lower education	910 (31%)	623 (38%)
Medium education	1696 (58%)	898 (55%)
High education	330 (11%)	108 (7%)
Anthropometric measures		
Body mass index, kg/m^2^	25 (23–28)	29 (26–32)
Waist circumference, cm	91 (83–99)	104 (96–111)
Blood pressure		
Systolic blood pressure, mmHg	125 (120–140)	140 (130–150)
Diastolic blood pressure, mmHg	80 (70–80)	80 (80–90)
Laboratory values		
Total cholesterol, mg/dL	224 (199–252)	217 (188–248)
HDL cholesterol, mg/dL	61 (51–72)	49 (41–58)
Fasting glucose, mg/dL	93 (87–98)	103 (97–112)
HbA1c, %	5.4 (5.2–5.6)	5.7 (5.4–5.9)
Lifestyle factors		
Ever smoking	1418 (46%)	927 (52%)
Alcohol consumption		
<2 drinks/day	2666 (90%)	1427 (86%)
≥2 drinks/day	284 (10%)	231 (14%)
Physical activity (LS7)		
Poor (<1 h/week)	309 (13%)	254 (20%)
Intermediate	1788 (75%)	906 (71%)
Ideal (≥3 h/week)	280 (12%)	125 (10%)
Diet quality (Life's simple 7)		
Poor diet	239 (8%)	194 (12%)
Intermediate diet	1757 (60%)	984 (59%)
Ideal diet	954 (32%)	480 (29%)
Family history		
Positive family history of CRC	388 (13%)	157 (9%)
Metabolic syndrome components		
ATP III criteria met		
Abdominal obesity	949 (31%)	1412 (79%)
Elevated triglycerides	288 (9%)	1260 (70%)
Low HDL cholesterol	195 (6%)	1025 (57%)
High blood pressure	1658 (53%)	1627 (91%)
Elevated fasting glucose	598 (19%)	1200 (67%)
Additional metabolic markers		
HOMA‐IR >2.5	396 (13%)	949 (53%)
IDF metabolic syndrome	359 (12%)	1672 (93%)

*Note*: Data are presented as median (interquartile range) for continuous variables and No. (%) for categorical variables. MetS was defined according to Adult Treatment Panel III criteria as the presence of ≥3 components: abdominal obesity, elevated triglycerides, low HDL cholesterol, hypertension or elevated fasting glucose.

Abbreviations: CRC, colorectal cancer; HbA1c, haemoglobin A1c; HDL, high‐density lipoprotein.

In the exploratory leptin subsample (*N* = 602), participants with ATP III MetS (*n* = 169) were older and more frequently male and exhibited a higher cardiometabolic burden, including higher BMI, waist circumference, blood pressure, fasting glucose/HbA1c and lower HDL cholesterol (Table S2). Median leptin concentrations were higher in participants with MetS (13.46 ng/mL) than in those without MetS (7.65 ng/mL). Advanced lesions and any adenoma were more frequent in the MetS subgroup within the leptin sample (advanced lesions 17% vs. 9%; any adenoma 52% vs. 38%; Table S2).

Advanced lesions (advanced adenoma or colorectal cancer) were detected in 389 participants (7.95%). Prevalence was higher among participants with MetS (9.7%; 173/1790) than among those without MetS (7.0%; 216/3101; *p* < 0.001) (Table 2). Any adenoma was also more common in the MetS group (38.3% vs. 29.4%; *p* < 0.001), accompanied by a higher adenoma burden across count categories and more frequent proximal, distal and rectal adenomas; similar location patterns were observed for advanced adenomas (Table 2). CRC was rare and did not differ significantly between groups (0.8% vs. 1.1%; *p* = 0.36) (Table 2).

**TABLE 2:** Adenoma outcomes by metabolic syndrome status.

Characteristic	No MetS (<3 criteria)	MetS (≥3 criteria)	*p* value
	*N* = 3101	*N* = 1790	
Primary outcome			
Advanced lesions	216 (7.0)	173 (9.7)	**<0.001**
Secondary outcomes			
Any adenoma	912 (29.4)	686 (38.3)	**<0.001**
Number of adenomas			
No adenoma	2204 (71.1)	1107 (61.8)	**<0.001**
One adenoma	567 (18.3)	401 (22.4)	
Two adenomas	198 (6.4)	146 (8.2)	
Three adenomas	80 (2.6)	71 (4.0)	
Four adenomas	30 (1.0)	24 (1.3)	
Five adenomas	10 (0.3)	21 (1.2)	
Six adenomas	5 (0.2)	9 (0.5)	
Seven adenomas	0 (0.0)	5 (0.3)	
Eight adenomas	2 (0.1)	3 (0.2)	
Nine adenomas	2 (0.1)	1 (0.1)	
≥10 adenomas	3 (0.1)	2 (0.1)	
Adenoma classification			
No neoplasia	2189 (70.6)	1104 (61.7)	**<0.001**
Non‐advanced adenoma	696 (22.4)	513 (28.7)	
Advanced adenoma	216 (7.0)	173 (9.7)	
Number of advanced adenomas			
No advanced adenoma	2896 (93.4)	1629 (91.0)	**0.033**
One advanced adenoma	171 (5.5)	137 (7.7)	
Two advanced adenomas	21 (0.7)	18 (1.0)	
Three advanced adenomas	9 (0.3)	3 (0.2)	
Four advanced adenomas	2 (0.1)	1 (0.1)	
Five advanced adenomas	0 (0.0)	1 (0.1)	
Six advanced adenomas	0 (0.0)	1 (0.1)	
≥10 advanced adenomas	1 (0.0)	0 (0.0)	
Adenoma location			
Proximal colon	554 (17.9)	450 (25.1)	**<0.001**
Distal colon	383 (12.4)	324 (18.1)	**<0.001**
Rectum	135 (4.4)	107 (6.0)	**0.012**
Advanced adenoma location			
Proximal colon	118 (3.8)	105 (5.9)	**<0.001**
Distal colon	116 (3.7)	107 (6.0)	**<0.001**
Rectum	50 (1.6)	45 (2.5)	**0.028**
Other findings			
Colorectal cancer	25 (0.8)	19 (1.1)	0.36

*Note*: Data are presented as No. (%). *p* values are from χ^2^ tests. Advanced adenomas were defined as adenomas with ≥1 of the following features: diameter ≥ 10 mm, villous or tubulovillous histology, or high‐grade dysplasia. Bold values are statistically significant values (*p* value < 0.05).

Abbreviation: MetS, metabolic syndrome.

**FIGURE 1:**
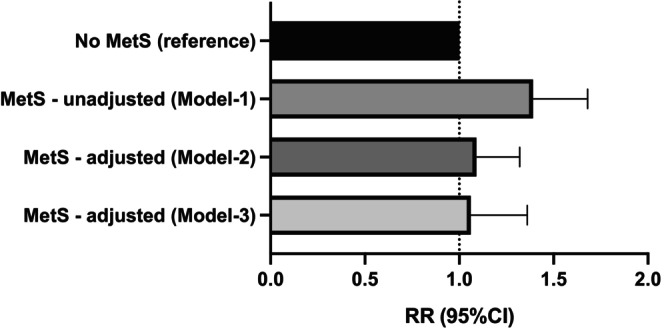
Association between metabolic syndrome and advanced adenomas. Risk ratios (RR) and 95% confidence intervals (CI) for advanced adenomas comparing participants with metabolic syndrome (MetS) to those without MetS (reference). Model 1 is unadjusted; Model 2 is adjusted for age and sex; and Model 3 is fully adjusted for age, sex, smoking status, alcohol consumption, family history of colorectal cancer, educational level, diet quality and physical activity. The vertical dashed line indicates the null value (RR = 1.0).

In unadjusted Poisson regression models, MetS was associated with a higher risk of advanced lesions (IRR: 1.388; 95% CI 1.146–1.680; *p* = 0.001) (Figure 1) (Table 3). This association attenuated after adjustment for age and sex (IRR: 1.089; 95% CI 0.896–1.323; *p* = 0.390) and remained non‐significant in the fully adjusted model (IRR: 1.062; 95% CI: 0.829–1.360; *p* = 0.635) (Table 3). Age was the strongest predictor of advanced lesions (fully adjusted: IRR 1.727 per decade; 95% CI: 1.531–1.949; *p* < 0.001), while female sex was consistently protective (IRR: 0.565; 95% CI: 0.436–0.732; *p* < 0.001) (Table 3). In the fully adjusted model, active smoking was associated with increased risk (IRR: 1.588; 95% CI: 1.164–2.166; *p* = 0.004), whereas alcohol consumption ≥2 drinks/day showed no evidence of association (IRR: 1.117; 95% CI: 0.750–1.664; *p* = 0.587) (Table 3). Family history of CRC showed a borderline association (IRR: 1.356; 95% CI: 0.983–1.870; *p* = 0.064), and high educational attainment was associated with a higher risk (IRR: 1.718; 95% CI: 1.127–2.620; *p* = 0.012), while diet quality and physical activity were not independently associated with advanced lesions in main‐effects models (Table 3). No consistent effect modification by age, sex, smoking status, alcohol consumption, family history, educational level or physical activity was observed (all interaction *p* > 0.05); an exploratory interaction with diet was observed for the poorest diet category (interaction IRR: 0.497; 95% CI: 0.266–0.932; *p* = 0.029) (Table 3).

**TABLE 3:** Association between metabolic syndrome and advanced colorectal lesions: Frequentist regression models.

Variable	Model 1 (unadjusted; *n* = 4891)	Model 2 (age/sex adjusted; *n* = 4891)	Model 3 (fully adjusted; *n* = 2819)	Interaction with MetS
	IRR (95% CI), *p* value	IRR (95% CI), *p* value	IRR (95% CI), *p* value	IRR (95% CI), *p* value
Metabolic syndrome	1.388 (1.146–1.680), **0.001**	1.089 (0.896–1.323), 0.390	1.062 (0.829–1.360), 0.635	—
Age (per decade)	—	1.509 (1.378–1.653), **<0.001**	1.727 (1.531–1.949), **<0.001**	0.880 (0.730–1.059), 0.176
Female sex	—	0.506 (0.412–0.620), **<0.001**	0.565 (0.436–0.732), **<0.001**	1.041 (0.686–1.579), 0.851
Smoking status				
Never smoker	—	—	Reference	Reference
Ex‐smoker	—	—	1.049 (0.794–1.385), 0.738	1.133 (0.696–1.845), 0.616
Active smoker	—	—	1.588 (1.164–2.166), **0.004**	1.040 (0.606–1.786), 0.886
Alcohol consumption				
<2 drinks/day	—	—	Reference	Reference
≥2 drinks/day	—	—	1.117 (0.750–1.664), 0.587	1.133 (0.640–2.007), 0.668
Family history of CRC	—	—	1.356 (0.983–1.870), 0.064	0.868 (0.482–1.563), 0.637
Educational level				
Lower education	—	—	Reference	Reference
Medium education	—	—	1.186 (0.885–1.591), 0.253	0.778 (0.499–1.214), 0.269
High education	—	—	1.718 (1.127–2.620), **0.012**	0.780 (0.392–1.553), 0.480
Diet quality				
Ideal diet	—	—	Reference	Reference
Intermediate diet	—	—	0.941 (0.724–1.224), 0.651	0.742 (0.476–1.155), 0.186
Poor diet	—	—	1.260 (0.805–1.973), 0.313	0.497 (0.266–0.932), **0.029**
Physical activity				
Poor	—	—	Reference	Reference
Intermediate	—	—	1.010 (0.699–1.461), 0.956	0.865 (0.478–1.565), 0.632
Ideal	—	—	1.203 (0.764–1.892), 0.425	1.497 (0.692–3.238), 0.305

*Note*: Data are presented as risk ratios (RR) with 95% confidence intervals (CI) estimated using Poisson regression with robust (sandwich) standard errors. Model 1 is unadjusted (*N* = 4891); Model 2 is adjusted for age and sex (*N* = 4891); and Model 3 is fully adjusted for age, sex, smoking status, alcohol consumption, family history of colorectal cancer, educational level, diet quality and physical activity (*N* = 2819). Bold values are statistically significant values (*p* value <0.05).

Abbreviations: CRC, colorectal cancer; MetS, metabolic syndrome.

In exploratory dose‐response analyses, the number of ATP III components was associated with advanced lesions in unadjusted models (IRR: 1.18 per additional component; 95% CI: 1.09–1.26; *p* < 0.001). This association attenuated completely after adjustment for age and sex (IRR: 1.03; 95% CI: 0.95–1.11; *p* = 0.54) and remained non‐significant in the fully adjusted model including lifestyle and sociodemographic covariates (*N* = 2819; IRR: 1.06; 95% CI: 0.96–1.17; *p* = 0.27), indicating that the crude gradient largely reflects confounding by age and sex.

Sensitivity analyses using the IDF MetS definition yielded concordant results. In unadjusted models, IDF MetS was associated with advanced lesions (IRR: 1.39; 95% CI: 1.15–1.69; *p* = 0.001), which attenuated after age/sex adjustment (IRR: 1.09; 95% CI: 0.90–1.32; *p* = 0.39) and in the fully adjusted model (IRR: 1.07; 95% CI: 0.83–1.37; *p* = 0.61). Similarly, HOMA‐IR as a binary indicator (>2.5) was not associated with advanced lesions in adjusted models. As a continuous exposure (per doubling), HOMA‐IR showed a borderline unadjusted association (IRR: 1.10; 95% CI: 1.00–1.21; *p* = 0.052) that was fully attenuated after age/sex adjustment (IRR: 1.04; 95% CI: 0.94–1.15; *p* = 0.46) and in the fully adjusted model (IRR: 1.03; 95% CI: 0.92–1.16; *p* = 0.61).

In the leptin subsample (*N* = 602; fully adjusted *N* = 411), leptin (per doubling) was not associated with advanced lesions in unadjusted (IRR: 0.85; 95% CI: 0.64–1.13; *p* = 0.26), age/sex‐adjusted (IRR: 0.96; 95% CI: 0.72–1.28; *p* = 0.77) or fully adjusted models (IRR: 0.95; 95% CI: 0.69–1.30; *p* = 0.74). In joint models including both leptin and MetS, leptin did not materially change the MetS estimates (fully adjusted MetS IRR: 1.08; 95% CI: 0.72–1.62; *p* = 0.71), supporting that leptin does not provide independent incremental risk information beyond the composite MetS definition.

**FIGURE 2:**
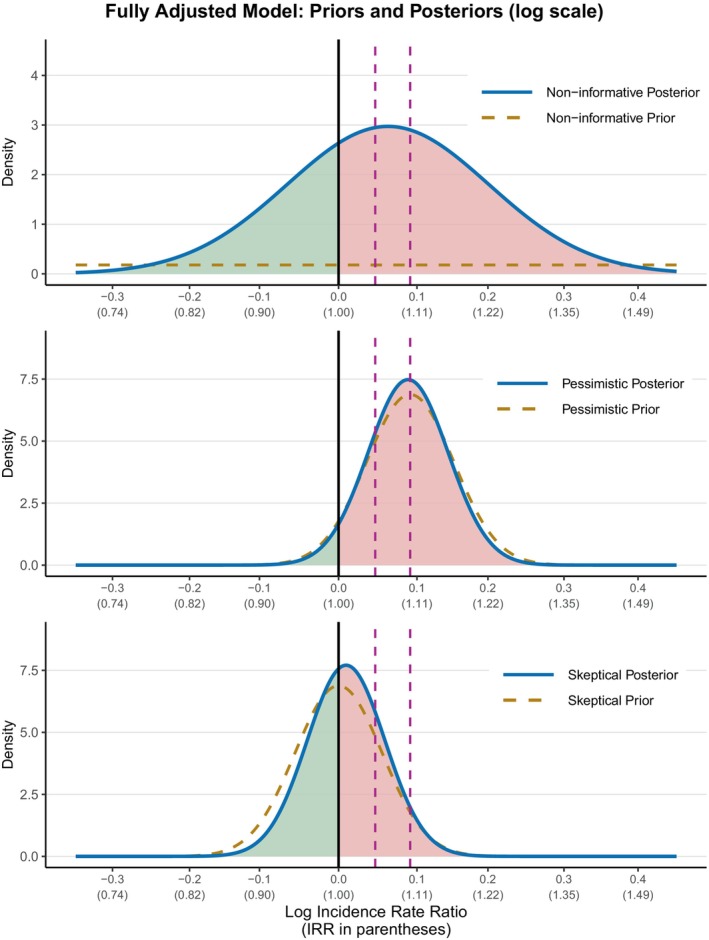
Priors and posterior distributions for the association of metabolic syndrome with advanced colorectal lesions in the fully adjusted model. Shown are prior (dashed lines) and posterior (solid lines) distributions for the log incidence rate ratio (IRR) of metabolic syndrome, derived from Bayesian Poisson regression. Results are displayed for a non‐informative prior (top), a pessimistic prior centred at ln (1.10) (middle) and a sceptical prior centred at 0 (bottom). Posterior densities are shaded red for IRR >1.0 and green for IRR <1.0. Vertical dashed lines indicate thresholds at ln (1.05) and ln (1.10). Probability boxes within each panel summarize the posterior probabilities for IRR exceeding these thresholds. The non‐informative prior appears nearly flat across the range shown, whereas the sceptical and pessimistic priors exert visible influence on the posterior.

Bayesian models corroborated the attenuation pattern observed in frequentist analyses while quantifying residual uncertainty (Table 4). In unadjusted analyses, posterior estimates supported an increased risk across prior strategies, including the non‐informative model (median IRR: 1.392; 95% CrI: 1.123–1.676; P (IRR > 1.10) = 99.2%). After age and sex adjustment, posterior distributions shifted toward the null (non‐informative median IRR: 1.087; 95% CrI: 0.884–1.320; P (IRR > 1.10) = 45.3%), and under sceptical priors centred on no effect the age‐ and sex‐adjusted median IRR was 1.023 (95% CrI: 0.924–1.125), with a high probability that the effect was within ±10% of the null (P (equivalence ±10%) = 91.5%). In fully adjusted models, posterior distributions concentrated further near no effect: under non‐informative priors the median IRR was 1.068 (95% CrI: 0.790–1.337; P (IRR > 1.10) = 41.5%), whereas sceptical priors yielded a median IRR of 1.010 (95% CrI: 0.911–1.116) with a 92.9% probability that the true effect lay within ±10% of the null. Under pessimistic priors anticipating a 10% increase, the fully adjusted median IRR was 1.097 (95% CrI: 0.993–1.224), corresponding to P (IRR > 1.05) = 79.0% and P (IRR > 1.10) = 48.2% (Figure 2) (Table 4).

**TABLE 4:** Bayesian sensitivity analysis: Association between metabolic syndrome and advanced colorectal lesions under different prior specifications.

Prior strategy	Median IRR	95% CrI	P (IRR > 1.0)	P (IRR > 1.05)	P (IRR > 1.10)	P (Equivalence ± 5%)	P (Equivalence ± 10%)
(A) Unadjusted models (*n* = 4891)							
Non‐informative	1.392	1.123–1.676	99.9%	99.7%	99.2%	0.3%	0.8%
Pessimistic	1.164	1.052–1.279	100.0%	97.9%	86.3%	2.1%	13.7%
Sceptical	1.081	0.983–1.196	94.2%	72.7%	36.7%	26.7%	63.3%
(B) Age and sex adjusted models (*n* = 4891)							
Non‐informative	1.087	0.884–1.320	78.6%	64.2%	45.3%	25.6%	50.7%
Pessimistic	1.099	0.989–1.206	96.7%	82.2%	48.7%	17.5%	51.3%
Sceptical	1.023	0.924–1.125	66.4%	29.9%	7.6%	62.0%	91.5%
(C) Fully adjusted models (*n* = 2819)							
Non‐informative	1.068	0.790–1.337	68.4%	55.1%	41.5%	24.8%	46.0%
Pessimistic	1.097	0.993–1.224	96.0%	79.0%	48.2%	20.6%	51.8%
Sceptical	1.010	0.911–1.116	57.5%	23.4%	5.2%	63.7%	92.9%

*Note*: Data are presented as median incidence rate ratios (IRR) with 95% CrcontributiI under three prior specifications: non‐informative (Normal [0, 100]), pessimistic (Normal [0.0953, 0.058^2^]) anticipating a 10% increased risk and sceptical (Normal [0, 0.058^2^]) centred on the null. Models were unadjusted (A), adjusted for age and sex (B) or fully adjusted for age, sex, smoking status, alcohol consumption, family history of colorectal cancer, educational level, diet quality and physical activity (C). Reported posterior probabilities indicate the likelihood of IRR exceeding specified thresholds (>1.0, >1.05, >1.10) or lying within equivalence regions (±5% or ±10% of the null).

As a sensitivity analysis, we examined whether individual ATP III components showed independent associations with advanced lesions among participants excluded from the primary analysis due to incomplete MetS data (*n* = 1477). Among these participants, component‐specific associations were likewise not robust after multivariable adjustment. Fully adjusted component‐specific estimates were non‐significant, including abdominal obesity (IRR: 1.957; *p* = 0.082), elevated triglycerides (IRR: 0.871; *p* = 0.642), low HDL cholesterol (IRR: 1.102; *p* = 0.758), high blood pressure (IRR: 1.564; *p* = 0.363) and elevated fasting glucose (IRR: 1.010; *p* = 0.978), supporting that component‐level signals were not consistently reproducible outside the main analytic cohort.

## DISCUSSION

In this large cross‐sectional screening cohort, MetS was associated with a higher prevalence of colorectal adenomas in unadjusted analyses. However, this relationship was fully attenuated after adjustment for age and sex, the two most established risk factors for colorectal neoplasia [4, 5, 19]. These findings suggest that the crude MetS‐adenoma association reflects demographic confounding rather than an independent metabolic effect. The close parallel between MetS and adenoma risk may reflect shared biological determinants rather than a causal link. Both conditions show strong age and sex dependencies, which are at least partly explained by hormonal and metabolic changes across the lifespan. In men, lower testosterone and sex hormone‐binding globulin (SHBG) concentrations are associated with both MetS and increased colorectal neoplasia risk, while in women, the menopausal transition markedly affects metabolic and neoplastic risk profiles [20, 21]. Adjusting for age and sex therefore accounts for these shared determinants, leaving little evidence for a residual, independent contribution of MetS to adenoma formation.

The marked attenuation of risk after demographic adjustment likely reflects the parallel age and sex dependencies of both conditions. MetS prevalence rises steeply with age, particularly among men and postmenopausal women, while adenoma risk increases almost exponentially with advancing age [20, 21, 22]. Similarly, male sex is an independent risk factor for MetS and colorectal neoplasia [5, 23, 24, 25]. The crude association observed in unadjusted models therefore appears to be explained by these shared demographic determinants rather than causal metabolic pathways. Sex‐related biology plausibly underlies the parallel age‐ and sex‐dependencies of both MetS and adenoma risk, including androgen/SHBG dynamics in men and menopausal transition in women. Our adjustment for age and sex therefore targets shared determinants rather than metabolic pathways per se. The absence of a residual MetS signal after adjustment suggests that any independent metabolic contribution to advanced adenomas is small relative to demographic effects.

Our exploratory analysis of leptin, a key adipokine linking adiposity and metabolic dysfunction, further supports this interpretation. Although leptin has been implicated in experimental models of tumorigenesis through pro‐inflammatory and pro‐angiogenic pathways, circulating leptin concentrations were not associated with advanced adenomas in our cohort. Adjustment for leptin did not materially change the null association between MetS and adenoma risk, arguing against a meaningful mediating role in colorectal carcinogenesis.

Bayesian analyses reinforced these conclusions. Unlike frequentist approaches, which focus on *p* values and dichotomous significance testing, Bayesian models allow the incorporation of prior knowledge and the direct estimation of the probability of clinically meaningful effects. In our study, non‐informative priors suggested considerable uncertainty, but sceptical priors centred on the null indicated a high probability of equivalence within ±10% of no effect. This analytic framework not only quantifies effect size and uncertainty more intuitively but also helps distinguish between statistical significance and clinical relevance, an important consideration in the context of population‐level screening policies.

Several biological mechanisms could theoretically link MetS to colorectal carcinogenesis, including insulin resistance, chronic inflammation and alterations of the gut microbiome [26, 27]. Beyond the composite MetS definition, adipokines may provide mechanistic resolution. Leptin is a key adipokine reflecting adiposity and metabolic dysfunction and has been implicated in pro‐inflammatory and pro‐proliferative signalling relevant to colorectal tumour biology. Given inconsistent clinical evidence, we examined leptin exploratorily to assess whether circulating leptin is associated with advanced colorectal lesions and whether it modifies or complements MetS‐based risk estimates. However, the absence of an independent MetS effect in this well‐characterized screening cohort, together with the lack of association for leptin, suggests that such mechanisms do not translate into clinically meaningful increases in adenoma risk once demographic factors are considered. Any potential residual effects are likely small and overshadowed by the overwhelming influence of age and sex.

These results do not support the inclusion of MetS as a criterion for CRC screening risk stratification. While individuals with MetS have a higher crude adenoma prevalence, this appears attributable to their demographic characteristics rather than metabolic dysfunction per se. Screening strategies should therefore continue to prioritize established factors such as age and sex, which remain the dominant drivers of risk.

This study has limitations. Its cross‐sectional design precludes causal inference, and residual confounding by unmeasured factors cannot be ruled out. The high prevalence of MetS in this cohort may indicate selection toward individuals with a higher cardiometabolic burden. Colonoscopy quality indicators, such as adenoma detection rate or bowel preparation quality, were not uniformly available and should be considered when interpreting absolute yield estimates. Symptoms were not systematically captured in the registry; thus, while procedures were conducted under a screening indication, some participants may have had non‐specific gastrointestinal complaints at the time of referral or self‐enrolment. This may limit inference regarding strictly asymptomatic screening populations.

Our analysis relied on complete‐case restriction, excluding 23% of the source population due to missing data in MetS components or covariates. While this approach limits statistical power, several lines of evidence suggest it does not introduce meaningful bias. The prevalence of advanced lesions was identical between included and excluded participants (8.0% vs. 8.0%; Table S1), and our substantive conclusions regarding the null association were consistent across multiple independent analytical approaches including dose‐response modelling, Bayesian inference, and sensitivity analyses using alternative exposure definitions. Missing data in SAKKOPI occurred primarily in structured clusters (e.g. complete laboratory panels) reflecting operational characteristics of opportunistic screening rather than participant‐level mechanisms amenable to multiple imputation. Nevertheless, we cannot entirely exclude the possibility of residual selection bias, and confirmation in prospective studies with complete planned ascertainment would be valuable.

Finally, the leptin analysis was restricted to a smaller subsample with available assays, resulting in wide confidence intervals and limited statistical power for exploratory analyses.

## CONCLUSIONS

In this large screening cohort, the crude association between MetS and colorectal adenomas was explained by age and sex. Leptin, a key adipokine mechanistically linked to obesity, showed no independent association with advanced adenomas. Bayesian analyses confirmed the lack of clinically meaningful effects, with a high probability of practical equivalence to the null. These findings argue against the use of MetS as an independent criterion for CRC screening risk stratification and reinforce the primacy of age and sex as the principal determinants of adenoma risk.

## AUTHOR CONTRIBUTIONS

**Franz Singhartinger:** Conceptualization; writing – original draft. **Georg Semmler:** Investigation; writing – review and editing. **Vera Paar:** Investigation; writing – review and editing. **Michael Lichtenauer:** Investigation; resources; writing – review and editing. **Andreas Völkerer:** Investigation; resources; writing – review and editing. **Josef Holzinger:** Investigation; writing – review and editing. **Mathias Ausserwinkler:** Investigation; writing – review and editing. **Maria Flamm:** Investigation; writing – review and editing. **Elmar Aigner:** Resources; writing – review and editing. **Christian Datz:** Investigation; data curation; resources; project administration. **Bernhard Wernly:** Conceptualization; investigation; data curation; resources; project administration; writing – original draft.

## FUNDING INFORMATION

No funding was received for this study.

## CONFLICT OF INTEREST STATEMENT

None declared.

## ETHICS STATEMENT

The local Ethics Committee for the province of Salzburg approved the study protocol (approval no. 415‐E/1262). Written informed consent was obtained from every participant.

## Supporting information


Table S1

Table S2

## Data Availability

The data that support the findings of this study are available on request from the corresponding author. The data are not publicly available due to privacy or ethical restrictions.
