# Supplementary material for: Metabolic syndrome and risk of advanced colorectal adenomas in a screening population: Frequentist and Bayesian analyses
Source: Colorectal Dis. 2026 Mar 19;28(3):e70424. doi: 10.1111/codi.70424 (PMC13003009; doi:10.1111/codi.70424)
Supplement: Supplementary file 1 — Table S1 [file CODI-28-0-s002.docx]

| **Supplemental Table S1:**  Comparison of Included and Excluded Participants |  |  |  |
| --- | --- | --- | --- |
|  |  |  |  |
| Characteristic | Excluded (N=1,477) | Included (N=4,891) | P Value |
| Demographics |  |  |  |
| Age, years | 61 (55-68) | 57 (51-65) | <0.001 |
| Male sex | 52% (550) | 52% (2,520) | 0.66 |
| Educational level |  |  | <0.001 |
| Lower education | 65% (615) | 34% (1,533) |  |
| Medium education | 33% (317) | 57% (2,594) |  |
| High education | 2% (19) | 10% (438) |  |
| Anthropometric measures |  |  |  |
| Body mass index, kg/m² | 28 (25-32) | 26 (24-29) | <0.001 |
| Waist circumference, cm | 102 (93-111) | 96 (87-104) | <0.001 |
| Blood pressure |  |  |  |
| Systolic blood pressure, mmHg | 140 (120-150) | 130 (120-140) | <0.001 |
| Diastolic blood pressure, mmHg | 80 (80-90) | 80 (75-87) | <0.001 |
| Laboratory values |  |  |  |
| Total cholesterol, mg/dL | 203 (173-232) | 222 (195-251) | <0.001 |
| HDL cholesterol, mg/dL | 54 (44-66) | 56 (47-67) | 0.001 |
| Fasting glucose, mg/dL | 106 (95-130) | 96 (90-104) | <0.001 |
| HbA1c, % | 5.7 (5.4-6.1) | 5.5 (5.2-5.8) | <0.001 |
| Lifestyle factors |  |  |  |
| Ever smoking | 36% (531) | 48% (2,345) | <0.001 |
| Alcohol consumption |  |  | <0.001 |
| <2 drinks/day | 76% (743) | 89% (4,093) |  |
| ≥2 drinks/day | 24% (238) | 11% (515) |  |
| Physical activity (LS7) |  |  | <0.001 |
| Poor (<1 hour/week) | 29% (326) | 15% (563) |  |
| Intermediate | 67% (753) | 74% (2,694) |  |
| Ideal (≥3 hours/week) | 4% (45) | 11% (405) |  |
| Diet quality (Life's Simple 7) |  |  | 0.020 |
| Poor diet | 12% (116) | 9% (433) |  |
| Intermediate diet | 60% (592) | 59% (2,741) |  |
| Ideal diet | 28% (273) | 31% (1,434) |  |
| Family history |  |  |  |
| Positive family history of CRC | 11% (120) | 11% (545) | 0.81 |
| Metabolic syndrome components |  |  |  |
| ATP III metabolic syndrome | 63% (659) | 37% (1,790) | <0.001 |
| IDF metabolic syndrome | 67% (698) | 42% (2,031) | <0.001 |
| Individual ATP III components |  |  |  |
| Abdominal obesity | 54% (797) | 48% (2,361) | <0.001 |
| Elevated triglycerides | 50% (528) | 32% (1,548) | <0.001 |
| Low HDL cholesterol | 41% (423) | 25% (1,220) | <0.001 |
| High blood pressure | 78% (820) | 67% (3,285) | <0.001 |
| Elevated fasting glucose | 71% (869) | 37% (1,798) | <0.001 |
| HOMA-IR >2.5 | 100% (1) | 27% (1,345) | 0.10 |
| Colonoscopy findings |  |  |  |
| Advanced lesions | 8% (81) | 8% (389) | 0.68 |
| Any adenoma | 31% (329) | 33% (1,598) | 0.38 |
| Adenoma location |  |  |  |
| Proximal colon | 20% (212) | 21% (1,004) | 0.78 |
| Distal colon | 15% (159) | 14% (707) | 0.58 |
| Rectum | 5% (51) | 5% (242) | 0.89 |
| Advanced adenoma location |  |  |  |
| Proximal colon | 4% (45) | 5% (223) | 0.69 |
| Distal colon | 4% (46) | 5% (223) | 0.79 |
| Rectum | 2% (24) | 2% (95) | 0.48 |
| Colorectal cancer | 1% (9) | 1% (44) | 0.89 |
