# Supplementary material for: Metabolic syndrome and risk of advanced colorectal adenomas in a screening population: Frequentist and Bayesian analyses
Source: Colorectal Dis. 2026 Mar 19;28(3):e70424. doi: 10.1111/codi.70424 (PMC13003009; doi:10.1111/codi.70424)
Supplement: Supplementary file 2 — Table S2 [file CODI-28-0-s001.docx]

| **Supplemental Table S2:** Characteristics of Participants with Available Leptin Measurements Stratified by Metabolic Syndrome Status |  |  |  |
| --- | --- | --- | --- |
|  |  |  |  |
| Characteristic | No MetS (N=433) | MetS (N=169) | P Value |
| Demographics |  |  |  |
| Age, years | 56 (52-61) | 60 (55-67) | <0.001 |
| Male sex | 45% (193) | 61% (103) | <0.001 |
| Educational level |  |  | <0.001 |
| Lower education | 8% (35) | 17% (28) |  |
| Medium education | 71% (302) | 72% (120) |  |
| High education | 20% (86) | 11% (18) |  |
| Anthropometric measures |  |  |  |
| Body mass index, kg/m² | 25 (23-28) | 29 (26-32) | <0.001 |
| Waist circumference, cm | 88 (81-96) | 102 (94-109) | <0.001 |
| Blood pressure |  |  |  |
| Systolic blood pressure, mmHg | 135 (124-145) | 140 (137-154) | <0.001 |
| Diastolic blood pressure, mmHg | 80 (75-89) | 85 (80-90) | <0.001 |
| Laboratory values |  |  |  |
| Total cholesterol, mg/dL | 229 (204-256) | 218 (190-246) | 0.002 |
| HDL cholesterol, mg/dL | 62 (53-74) | 50 (41-58) | <0.001 |
| Fasting glucose, mg/dL | 92 (86-97) | 104 (96-110) | <0.001 |
| HbA1c, % | 5.3 (5.1-5.5) | 5.5 (5.4-5.8) | <0.001 |
| Leptin, ng/mL | 7.65 (4.25-13.81) | 13.46 (7.61-22.32) | <0.001 |
| Lifestyle factors |  |  |  |
| Ever smoking | 40% (173) | 57% (96) | <0.001 |
| Alcohol consumption |  |  | 0.059 |
| <2 drinks/day | 97% (413) | 93% (151) |  |
| ≥2 drinks/day | 3% (14) | 7% (11) |  |
| Physical activity (LS7) |  |  | 0.60 |
| Poor (<1 hour/week) | 5% (14) | 7% (8) |  |
| Intermediate | 82% (250) | 78% (93) |  |
| Ideal (≥3 hours/week) | 14% (42) | 15% (18) |  |
| Diet quality (Life's Simple 7) |  |  | 0.18 |
| Poor diet | 4% (19) | 7% (12) |  |
| Intermediate diet | 56% (241) | 60% (97) |  |
| Ideal diet | 39% (167) | 33% (53) |  |
| Family history |  |  |  |
| Positive family history of CRC | 15% (67) | 7% (12) | 0.006 |
| Metabolic syndrome components |  |  |  |
| ATP III metabolic syndrome | 0% (0) | 100% (169) | <0.001 |
| IDF metabolic syndrome | 12% (51) | 92% (155) | <0.001 |
| Individual ATP III components |  |  |  |
| Abdominal obesity | 23% (101) | 72% (121) | <0.001 |
| Elevated triglycerides | 9% (41) | 72% (121) | <0.001 |
| Low HDL cholesterol | 3% (13) | 51% (86) | <0.001 |
| High blood pressure | 69% (297) | 96% (163) | <0.001 |
| Elevated fasting glucose | 15% (65) | 65% (110) | <0.001 |
| HOMA-IR >2.5 | 9% (38) | 49% (83) | <0.001 |
| Colonoscopy findings |  |  |  |
| Advanced lesions | 9% (40) | 17% (28) | 0.003 |
| Any adenoma | 38% (166) | 52% (88) | 0.002 |
| Adenoma location |  |  |  |
| Proximal colon | 20% (87) | 35% (59) | <0.001 |
| Distal colon | 17% (73) | 24% (40) | 0.055 |
| Rectum | 4% (19) | 7% (11) | 0.29 |
| Advanced adenoma location |  |  |  |
| Proximal colon | 5% (21) | 11% (18) | 0.009 |
| Distal colon | 4% (19) | 12% (20) | <0.001 |
| Rectum | 2% (7) | 3% (5) | 0.29 |
| Colorectal cancer | 1% (3) | 1% (2) | 0.55 |
